# An error in interpretation and real extraordinary electrocardiographic changes in patient with acute traumatic spondylolisthesis

**DOI:** 10.1186/s43044-022-00301-w

**Published:** 2022-09-03

**Authors:** Kadir Karacali, Damla Yalcinkaya, Bilal Canberk Ilhan, Mikail Yarlioglues

**Affiliations:** Department of Cardiology, Ankara Education and Research Hospital, 06340 Altindag, Ankara, Turkey

**Keywords:** Acute cervical trauma, ECG, ECG display format

## Abstract

**Background:**

Acute cervical spinal trauma may lead to cardiac effects by influencing cardiac sympathetic preganglionic fibers. Some of these effects, which are vital, may occur in ECG.

**Case presentation:**

A 52-year-old female patient admitted to the emergency department with acute traumatic spondylolisthesis at *C*6-*C*7 level and paraplegia. Positive QRS complex, ST segment depressions and prolongation of QTc interval were observed on ECG according to sudden autonomic disruption because of sympathetic nerve compression. It was mentioned that changes in QRS complex axis was normal which was dependent to the ECG display format of Cabrera sequence used differently from the classical system. After surgical correction, evident ST depressions were recovered and QTc intervalwas narrowed but still prolonged in control ECG.

**Conclusions:**

Autonomic dysfunction can lead to extraordinary electrocardiographic presentation including widespread ST depressions with prolonged QTc interval. However, when evaluating the changes in the ECG, attention should be paid to ECG display format to avoid errors in interpretation.

**Supplementary Information:**

The online version contains supplementary material available at 10.1186/s43044-022-00301-w.

## Background

In a previous case report of a patient with acute cervical spinal trauma, ECG demonstrated sinus tachycardia, first-degree atrioventricular block, ST-segment depression in anterior and inferior leads, right bundle-branch block and prolonged QT interval [1]. Cardiac sympathetic preganglionic fibers originate from the superior cervical, middle cervical and most commonly from the cervicothoracic and thoracic ganglia. The middle cervical ganglion is located at the *C*6 level and the cervicothoracic ganglion at the *C*7 level [2]. Therefore, spondylolisthesis at *C*6–*C*7 level can cause sympathetic nerve compression with sudden autonomic disruption. Autonomic dysfunction can lead to extraordinary electrocardiographic presentation including widespread ST depressions with prolonged QTc interval.

However, some special conditions should be considered while interpreting the ECG in terms of extraordinary findings. Several factors can be considered as the reason of these changes including incorrect lead placement, dextrocardia, acute coronary syndrome, and ECG display format. Here we report a patient with cervical trauma and prominent ECG changes.

## Case presentation

**Fig. 1:**
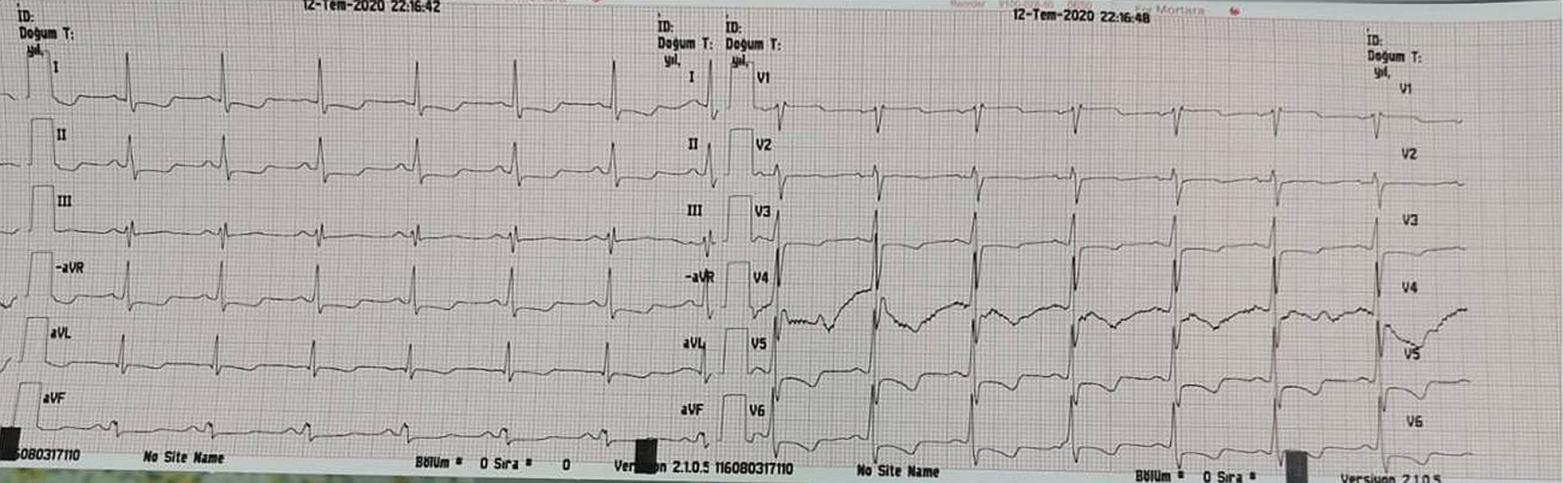
The first preoperative electrocardiography

**Fig. 2:**
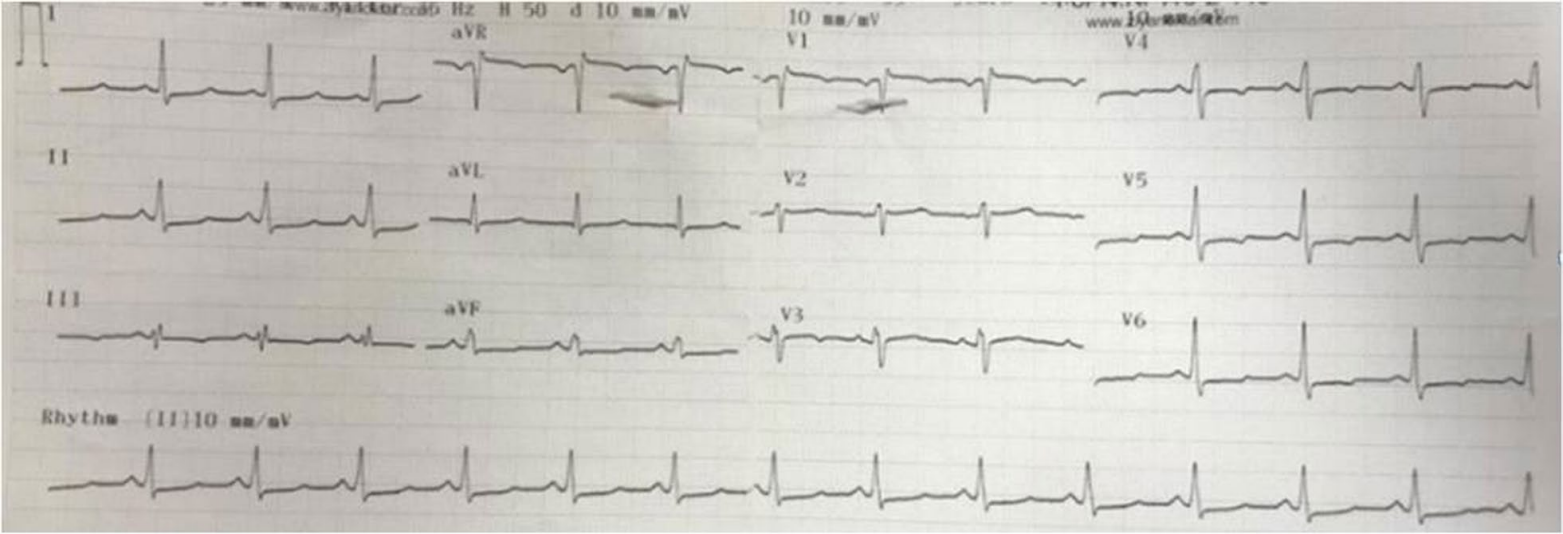
Preoperative control electrocardiography

A 52-year-old female patient admitted to the emergency department with fall from height. She had developed paraplegia. Her heart rate was 70 beats/min with blood pressure of 110/70 mmHg. Magnetic resonance imaging was performed emergently and it revealed spondylolisthesis at *C*6–*C*7 level (Additional file 1). She was immediately taken into surgery room. During preoperative evaluation and preparation for anesthesia, tall R wave in aVR lead and widespread ST segment depressions were noticed on ECG. Urgent cardiology consultation was requested with pre-diagnosis of acute coronary syndrome. At the bedside evaluation of the patient, we determined that there was positive QRS complex, upright P and tall R wave in aVR lead and widespread ST segment depressions at all derivations with prolonged corrected QT (QTc) interval (522 ms) on ECG (Fig. 1).First, we excluded incorrect lead placement by repeating the ECG on our witness. Then, we also excluded dextrocardia by auscultation and palpation of strong heart beat at normal anatomic localization in the precordium at the level of 5th left intercostal space, medial to the left midclavicular line. Right after that, we had mentioned the difference in the ECG display format. Later, we had performed ECG at standard display format using another machine. It revealed normal QRS axis with negative P and T waves in aVR. This time, ST segment elevation in leads V1 and aVR attracted our attention (Fig. 2). Other evident abnormalities at the patient's ECG were prolongation of the QTc interval and widespread ST segment depression in all leads. Although, there were ST segment elevations in leads aVR and V1 on the preoperative control ECG, acute coronary syndrome requiring emergency intervention was not considered, since the patient was hemodynamically stable.

**Fig. 3:**
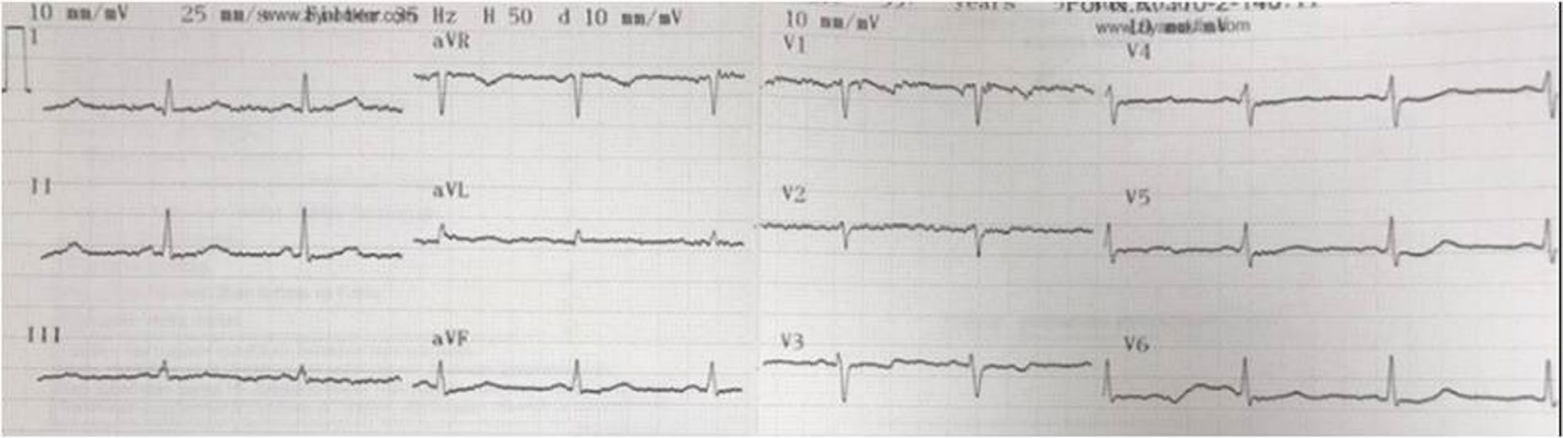
Postoperative control electrocardiography

It was decided to start surgery considering the patient’s stable hemodynamic and the necessity of an urgent intervention to spondylolisthesis. Surgical correction was achieved successfully. Postoperative control computed tomography showed correction of the displaced vertebra (Additional file 2). On the postoperative control ECG, evident ST depressions were recovered and QTc interval was narrowed but still prolonged (500 ms) (Fig. 3). Since the patient had cardiovascular-risk factors and previous ECG changes, coronary angiography was performed after the procedure and normal coronary arteries were observed (Additional file 3). At follow up, successful extubation and spontaneous breathing were achieved but paraplegia continued.

## Discussion

The primary evident abnormalities that took attention at the patient's ECG were positive QRS complex; upright P and tall R wave in lead aVR. In such cases, incorrect lead placement, dextrocardia, acute coronary syndrome, and an often-overlooked detail, which is the ECG display format, should be considered. The first steps in ECG readingis to check the aVR lead before starting to evaluate the whole. The aVR stands for the augmented vector where the positive electrode is on the right shoulder. It is a unipolar lead facing the right upper side of the heart. It also gives reciprocal information on the left lateral side of the heart, which is already covered by leads aVL, I, II, V5, and V6. All waves (P, QRS, and T) are negative in aVR in normal sinus rhythm, as all the depolarization waves diverge away from lead aVR [3]. Therefore, any difference in the QRS polarities suggests an abnormal situation as in our patient. We talk about possible reasons.

Incorrect limb lead placement is one of the most common reasons that should come to mind first. With LA and RA reversal, lead DI becomes inverted, lead aVR becomes positive and right axis deviation can be seen. With RA and LL reversal, leads I, II, III and aVF are all inverted and lead aVR becomes upright. AVR remains unchanged with LA and LL reversal [4].In contrast to dextrocardia, normal R-wave progression is present in the precordial leads.

Another reason that should primarily come to mind is dextrocardia. However, in dextrocardia, we should expect poor R wave progression in precordial leads with negative QRS complexes in lead I which were absent in our case [5].

One of the rare reasons other than those mentioned above is acute coronary syndrome. Especially in anterior myocardial infarction with ventricular aneurysm, we may see tall R waves in aVR, called Goldberger’s sign. However, it should be accompanied by persisted ST elevation in precordial leads [3].Butour patient had widespread ST depressions in the precordial leads.

**Fig. 4:**
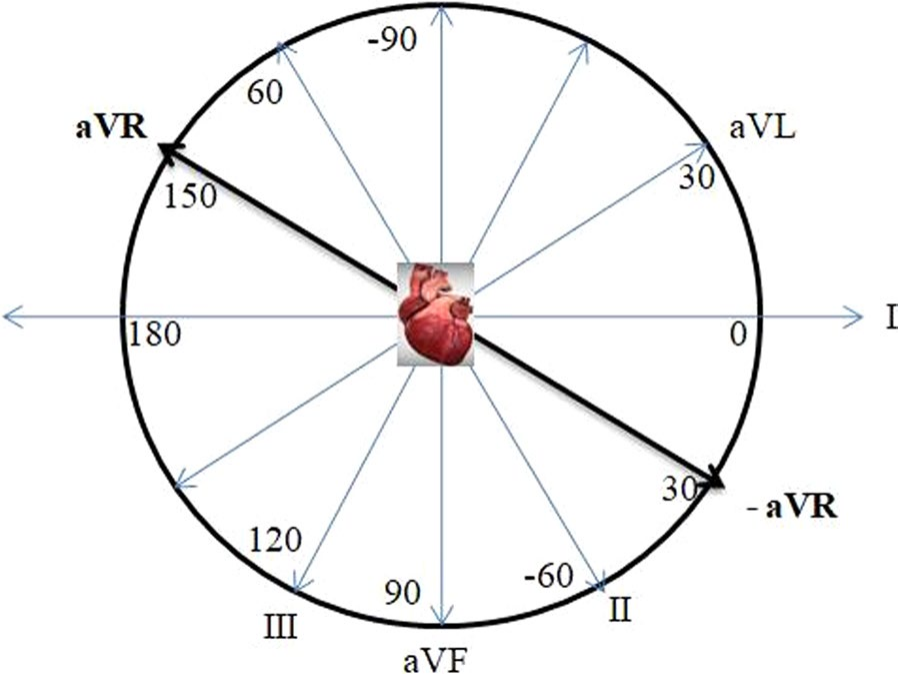
Diagram of the limb leads within the frontal plane in the Cabrera system

Another reason that can be often overlooked is ECG display format. The classical system is widely use in United States, most of European countries, Asia and Africa. In the classical system, the limb leads are displayed within the frontal plane by dual sequences as 60° intervals between lead I, lead II and lead III and 120° intervals between lead aVR, lead aVL and lead aVF. The lead aVR exits at − 150 ͦ in the frontal plane. However, the Cabrera system presents six anatomically ordered frontal plane leads. It reverses the polarity of lead aVR and presents the ECG complexes in the order of aVL, I, -aVR, II, aVF, III, respectively (Fig. 4) [6]. The lead -aVR exits at 30 ͦ in frontal plane. Thus, lead –aVR fills the gap between lead I and lead II in the coordinate system. While all depolarizations approach to lead -aVR, all waves (P, QRS, and T) are positive in aVR in normal sinus rhythm. Evaluation of leads aVL, I and -aVR together improves the diagnosis of acute lateral wall ischemia or infarction. In addition, using –aVR facilitates cardiac electrical axis determination. Cabrera system has been the routine display method in Sweden for many years. Routine use of the Cabrera sequence was recommended as an alternative by American Heart Association in their AHA/ACC/HRS scientific statements report [7]. It was suggested that manufacturers should be encouraged to offer this display as a routine option in new electrocardiographs. At present, all modern ECG machines can be easily switched from the standard system (aVR) to the Cabrera system (-aVR).

We admit that we had never used the Cabrera system before. We did not pay attention to the minus sign in front of the aVR statement indicating -aVR in the ECG paper at first look. First, we excluded incorrect lead placement and dextrocardia. Then we realized the difference in the ECG display format and repeated ECG at standard display format using another machine.

Finally, it was decided that ECG changes mentioned above might be related to autonomic dysfunction due to acute cervical trauma. In the heart, sympathetic stimulation shortens action potential duration and reduces transmural dispersion of repolarization on both atrial and ventricular myocytes while parasympathetic stimulation prolongs action potential duration and effective refractory period in the ventricles, but reduces the atrial effective refractory period augments spatial electrophysiological heterogeneity and promotes early after depolarization toward the end of phase 3 in the action potential in the atria. Thus, autonomic dysfunction leads to varying changes action potential that affect to all ECG components including QRS complex, ST segment, QT duration.

## Conclusions

Autonomic dysfunction can lead to extraordinary electrocardiographic presentation including widespread ST depressions with prolonged QTc interval. Early successful surgical correction can provide recovering of evident ST depressions and narrowing of QTc on ECG. However, when evaluating the changes in the ECG, attention should be paid to ECG display format to avoid errors in interpretation.

## Supplementary Information

**Additional file 1.** Magnetic resonance image showing spondylolisthesis at C6-C7 level.

**Additional file 2. **Postoperative control computed tomography showing correction of the displaced vertebra.

**Additional file 3. **Postoperative coronary angiography.

## Data Availability

The datasets used and/or analyses during the current study are available from the corresponding author on reasonable request.

## Abbreviations

ECG: Electrocardiography
QTc: Corrected QT
LA: Left arm
RA: Right arm
LL: Left leg

## References

[1] Mehta NJ, Mehta RN, Khan IA (2001). Electrocardiographic changes of acute cervical spinal cord trauma. Clin Cardiol.

[2] Yin Z, Yin J, Cai J, Sui T, Cao X (2015). Neuroanatomy and clinical analysis of the cervical sympathetic trunk and longus colli. J Biomed Res.

[3] Chenniappan M, Sankar RU, Saravanan K, Karthikeyan K (2013). Lead aVR–the neglected lead. J Assoc Phys India.

[4] Batchvarov VN, Malik M, Camm AJ (2007). Incorrect electrode cable connection during electrocardiographic recording. Europace.

[5] George A, Arumugham PS, Figueredo VM (2010). aVR - the forgotten lead. Exp Clin Cardiol.

[6] Lam A, Wagner GS, Pahlm O (2015). The classical versus the Cabrera presentation system for resting electrocardiography: impact on recognition and understanding of clinically important electrocardiographic changes. J Electrocardiol.

[7] Kligfield P, Gettes LS, Bailey JJ, Childers R, Deal BJ, Hancock EW, Josephson M, Mason JW, Okin P, Surawicz B, Wellens H (2007). Recommendations for the standardization and interpretation of the electrocardiogram: part I: The electrocardiogram and its technology: a scientific statement from the American Heart Association Electrocardiography and Arrhythmias Committee, Council on Clinical Cardiology; the American College of Cardiology Foundation; and the Heart Rhythm Society: endorsed by the International Society for Computerized Electrocardiology. Circulation.

