# Supplementary figures and images for: An error in interpretation and real extraordinary electrocardiographic changes in patient with acute traumatic spondylolisthesis
Source: Egypt Heart J. 2022 Sep 3;74:62. doi: 10.1186/s43044-022-00301-w (PMC9440963; doi:10.1186/s43044-022-00301-w)

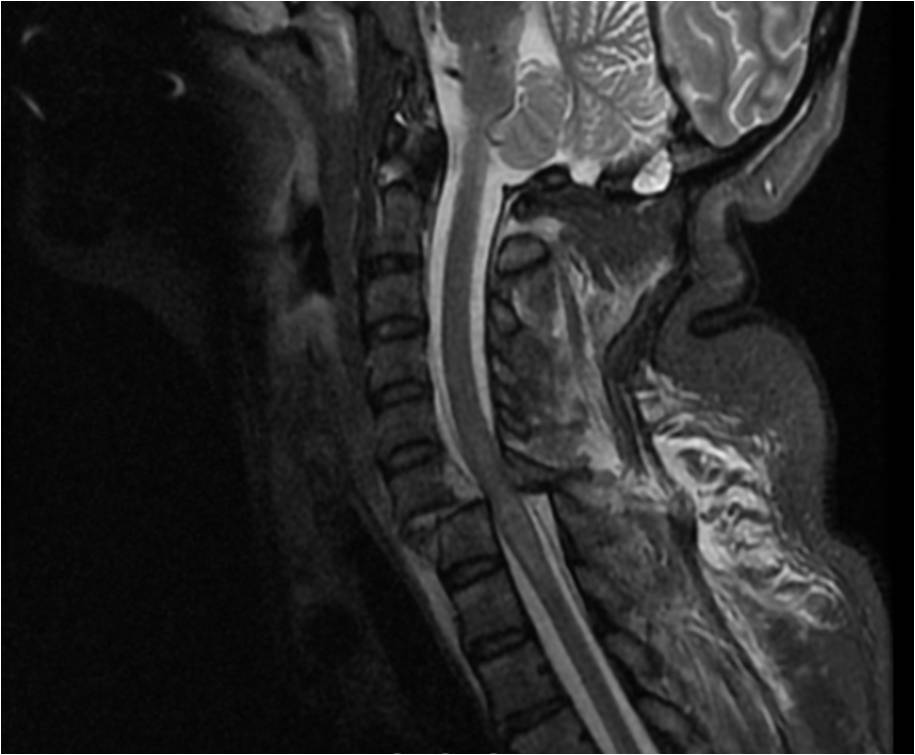

Supplement: Supplementary file 1 — Additional file 1. Magnetic resonance image showing spondylolisthesis at C6-C7 level. [file 43044_2022_301_MOESM1_ESM.jpg]

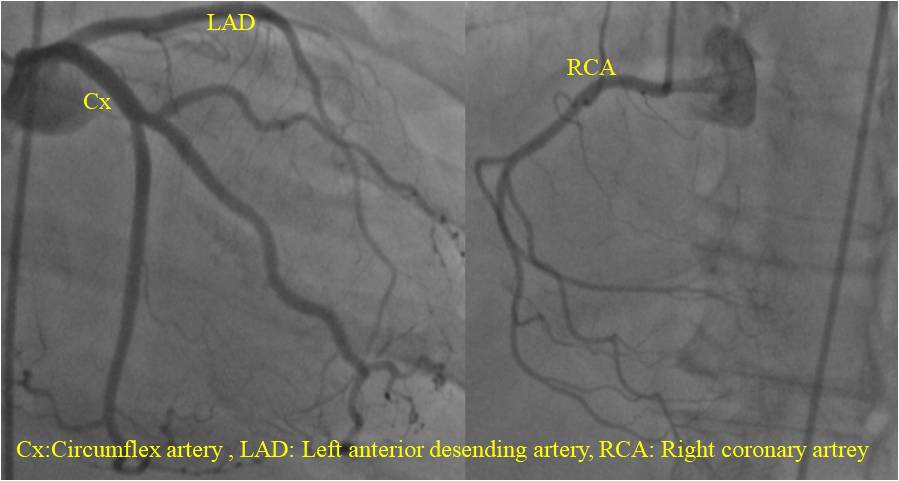

Supplement: Supplementary file 2 — Additional file 2. Postoperative control computed tomography showing correction of the displaced vertebra. [file 43044_2022_301_MOESM2_ESM.jpg]

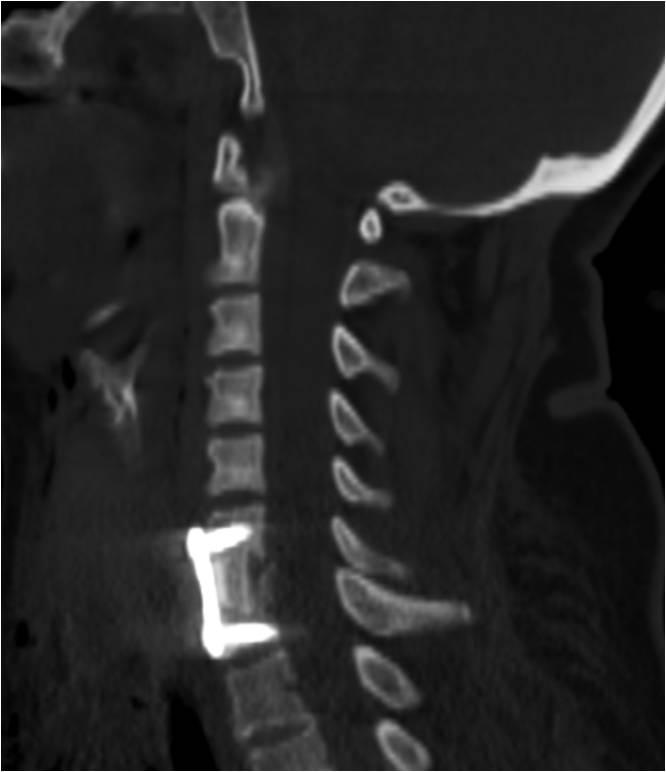

Supplement: Supplementary file 3 — Additional file 3. Postoperative coronary angiography. [file 43044_2022_301_MOESM3_ESM.jpg]
